# Monocyte and macrophage immunometabolism in atherosclerosis

**DOI:** 10.1007/s00281-017-0656-7

**Published:** 2017-10-02

**Authors:** Laszlo Groh, Samuel T. Keating, Leo A. B. Joosten, Mihai G. Netea, Niels P. Riksen

**Affiliations:** 1 Department of Internal Medicine (463) and Radboud Institute for Molecular Life Sciences (RIMLS), Radboud University Medical Center, PO Box 9101, 6500 HB Nijmegen, The Netherlands; 2 Department for Genomics and Immunoregulation, Life and Medical Sciences Institute (LIMES), University of Bonn, 53115 Bonn, Germany

**Keywords:** Atherosclerosis, Immunometabolism, Innate immune memory, Trained immunity, Epigenetic reprogramming

## Abstract

Atherosclerosis is characterized by chronic low grade inflammation of arteries that results in the development of lipid dense plaques. Chronic inflammation induced by Western-type diet is associated with the risk of developing atherosclerosis, and new insights shed light on the importance of metabolic and functional reprogramming in monocytes and macrophages for progression of atherosclerosis. This review aims to provide an overview of our current understanding into how the metabolic reprogramming of glucose, cholesterol, fatty acid, and amino acid metabolism in macrophages contributes to inflammation during atherosclerosis. Recent insights suggest that transcriptional and epigenetic adaptation within innate immune cells (termed *trained immunity*) play an important role in the pathogenesis of atherosclerosis. We propose that metabolic changes induced by pro-atherogenic lipoproteins partly mediate these changes in trained macrophages. Finally, we discuss the possibility of manipulating cellular metabolism of immune cells for targeted therapeutic intervention against atherosclerosis.

## Introduction

In most patients, acute myocardial infarction and stroke are the consequence of erosion or rupture of large artery atherosclerotic plaques and the subsequent local formation of an occluding thrombus. The risk to develop atherosclerosis is determined to a large extent by common cardiovascular risk factors such as smoking, dyslipidemia, hypertension, diabetes, and obesity. In addition, chronic inflammatory conditions, including rheumatoid arthritis and psoriasis, increase atherosclerotic cardiovascular risk independent from the traditional risk factors.

Atherosclerosis is characterized by a chronic non-resolving low-grade sterile inflammation of the arterial wall. It predominantly occurs at arterial sites of disturbed laminar flow, where subendothelial accumulation of apolipoprotein B-containing lipoproteins is the key initiating step. In the last two decades, macrophages have been identified as protagonists of atherosclerosis and its thrombotic complications [1]. Inflammatory macrophages are the most abundant immune cells within plaques, originating from circulating monocytes that bind activated endothelial cells and migrate into the intimal layer [2], as well as from local proliferation of resident macrophages [3]. Lesion formation is markedly reduced when circulating monocytes are prevented from binding to endothelial cells [4]. Within the plaque, macrophages orchestrate the progression of the atherosclerotic process by the uptake of oxidized low-density lipoprotein particles (oxLDL) and subsequent foam cell formation. In addition, macrophages respond with the production of a large variety of pro-atherogenic cytokines and chemokines upon stimulation with danger-associated molecular patterns, such as oxLDL and proteoglycans, which are present in the atherosclerotic milieu [5]. Finally, macrophages influence plaque stability by regulating collagen production and by the production of proteases such as matrix metalloproteinase [1].

Within plaques, macrophage phenotype is shaped largely by external stimuli. The traditional classification into pro-inflammatory M1 macrophages and anti-inflammatory M2 macrophages was reported to be an oversimplification of reality in which diverse activation signals drive a spectrum of macrophages [6]. Importantly, these distinct activation states have different energy requirements. Therefore, it is not surprising that the intracellular metabolism of these macrophages varies considerably. However, it has only recently emerged that the intracellular metabolic pathways not only follow the energy demands of the cells but also actually regulate the functional state of the cells in response to environmental cues, such as oxygen and nutrient availability, growth factors, and cytokines [7, 8]. This plasticity is a characteristic not only of local plaque macrophages but also of circulating monocytes and their bone marrow progenitors that are also exposed to pro-atherogenic stimuli such as lipoproteins, glucose, diet, and microbiota-derived substances. Complementing this view that the functional states of macrophages are shaped by tissue microenvironment is the recent finding that brief encounters of monocytes with pro-atherogenic stimuli can induce a long-lasting inflammatory monocyte/macrophage phenotype either in the bone marrow or in the circulation. This innate immune memory has recently been termed “trained innate immunity” [9, 10].

## Trained immunity as a novel mechanism of atherogenesis

The adaptive immune system, which consists of B and T cells, generates specific T cell clones and antibodies that target invading pathogens. This garners highly effective and specific protection against re-infection by the same pathogen. Traditionally, innate immune cells were resigned to the task of nonspecific elimination of the pathogen either by cellular mechanisms such as phagocytes or NK cells, or humoral mechanisms such as the complement system. However, a growing body of evidence demonstrated that monocytes and macrophages retain memories of past infections rendering them more or less responsive to re-challenge. This innate immune memory is comprised of a hyper-responsive phenotype termed *trained innate immunity* or *trained immunity* and a hypo-responsive phenotype termed *tolerance*. Tolerance is induced experimentally by subjecting monocytes to high doses of lipopolysaccharides (LPS). Upon maturation into macrophages, these tolerized cells are refractory to secondary stimulation. At the other end of the spectrum, trained immunity is exemplified by increased cell responsiveness after exposure to β-glucan [11], a component of the *Candida albicans* cell wall, or Bacillus Calmette-Guérin (BCG) [12]. Monocytes that come into contact with a moderate amount of these stimuli give rise to macrophages that mount an augmented response towards secondary stimulation. This trained phenotype is also induced in vivo by BCG vaccination in healthy subjects, with ex vivo stimulation of circulating peripheral blood mononuclear cells showing enhanced responsiveness for up to 3 months [12].

In the context of recurrent infections, trained immunity provides robust protection against re-infection. This is illustrated by the prevention of mortality in mice after lethal *C*. *albicans* challenge when the mice received BCG vaccination 2 weeks earlier [12, 13]. The leading cause of death in Western societies is no longer infectious diseases, due to the development of antibiotics and vaccinations. In contrast, cardiovascular disease has replaced infections as the major cause of death. The role of innate immune memory in propagating chronic inflammatory states, such as atherosclerosis, is under current and thorough investigation. In this context, calibration of innate immune cells for heightened inflammatory responses represents an added risk for the development and progression of atherosclerosis. Indeed, vaccination with BCG accelerates atherosclerosis in rabbits fed a cholesterol-rich diet [14]. However, other data suggest that hyperlipidemic mice fed a high-cholesterol diet had a delayed atherosclerotic plaque formation 6 weeks following BCG injection. It is important to realize, though, that in these mice, the BCG administration significantly lowered non-HDL cholesterol levels [15].

Specific to cardiovascular disease, trained immunity can also be induced by non-microbial pro-atherogenic stimuli, such as oxLDL and lipoprotein (a). Brief stimulation of isolated human monocytes to oxLDL induces a macrophage phenotype that responds with enhanced production of TNFα and IL-6 to re-stimulation with Toll-like receptor (TLR) 2 and TLR4 ligands and signaling through the PI3K and MAPK pathway [16]. Inhibition of histone methylation by the nonspecific methyltransferase inhibitor 5′-methylthioadenosine completely ablated the training induced by oxLDL suggesting that epigenetic reprogramming is important for oxLDL-induced trained immunity. Apart from increases in cytokine production, oxLDL-trained macrophages exhibited increased expression of the oxLDL recognition receptors CD36 and SR-A, as well as reduced expression of the cholesterol efflux transporters ABCA1 and ABCG1. Functionally, oxLDL-trained macrophages also exhibited greater oxLDL uptake, and an increased propensity to form foam cells. Other pro-atherogenic factors increased in oxLDL-trained macrophages were the chemokine MCP-1 as well as the collagenases MMP-2 and MMP-9, suggesting possible roles for oxLDL-trained macrophages in the progression of atherosclerosis pathogenesis and plaque destabilization. In addition to oxLDL, a pro-atherogenic-trained macrophage phenotype was obtained by brief stimulation to lipoprotein (a) [17]. These findings suggest that although trained immunity is beneficial in the context of recurrent infections, it might actually accelerate the development of atherosclerosis.

**Fig. 1:**
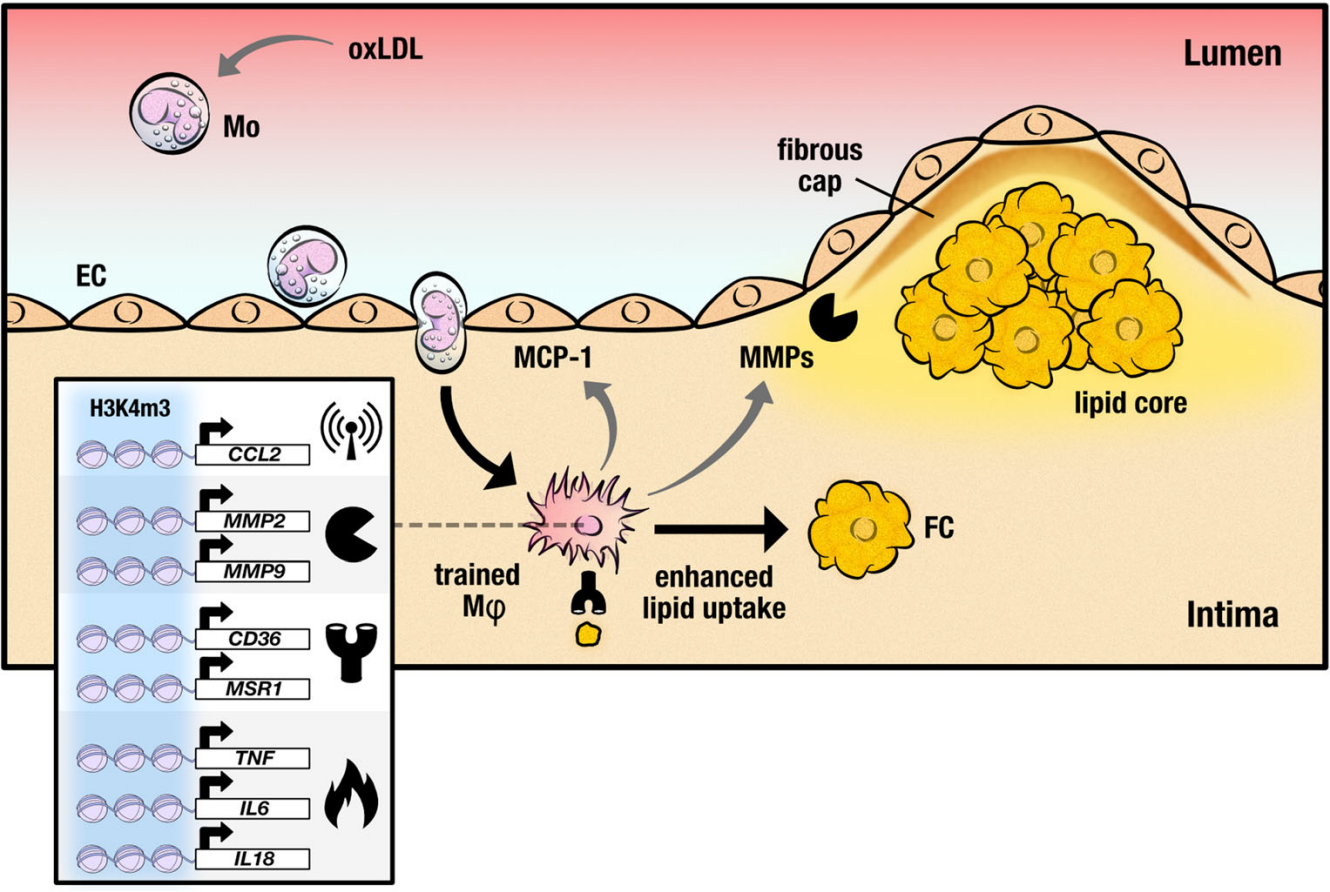
Trained macrophages drive atherosclerosis progression. Monocytes are recruited into the intima after binding to activated endothelial cells. Once in the intima, monocytes differentiate into macrophages. Trained monocytes show higher expression of *CCL2*, encoding monocyte chemoattractant protein 1 (MCP-1), which signals the recruitment of additional monocytes. Furthermore, trained macrophages produce high levels of pro-inflammatory cytokines such as TNF alpha, IL6, and IL18. Increased expression of lipid scavenging receptors *CD36* and *SR*-*A* enhances the gross uptake of modified lipids, generating foam cells which aggregate together in the lipid core. Plaque destabilization results from increased matrix metalloproteinase (MMP) production from pro-inflammatory macrophages, promoting degradation of the fibrous cap. These changes in gene expression are at least partly driven by the enrichment of H3 histones methylated at lysine 4 at regulatory promoters

In 2014, two landmark papers characterized the epigenetic and metabolic profile of macrophage innate immune memory [18, 19]. Epigenetic reprogramming at the level of histone modifications was identified as crucial intracellular mechanisms that determined the enhanced functional state following brief exposure of the cells to selected microorganisms and microbial products. Epigenetic analysis of several activating histone methylation and acetylation marks revealed distinct epigenetic signature characteristic of naïve, tolerant, or β-glucan-trained macrophages [19]. Training with oxLDL corresponds with enrichment of H3K4me3 at the promoters of *TNFA*, *IL6*, *MCP1*, *IL8*, *CD36*, *SR*-*A*, *MMP2*, and *MMP9* (Fig. 1) [16]. Gene ontology analysis showed that β-glucan training induces the expression of genes associated with central metabolism, most notably with the glycolysis and tricarboxylic acid cycle (TCA), spawning the hypothesis that metabolic reprogramming is also important for the functional fate of trained immunity [18].

Recent data suggests that monocytes with a “trained” phenotype are present in patients with atherosclerosis or associated risk factors. Monocytes isolated from subjects with elevated circulating levels of lipoprotein (a) showed a similar increased cytokine production capacity, which was associated with enhanced endothelial binding and transendothelial migration, and increased vascular wall inflammation in vivo, as measured with fluorodeoxyglucose positron emission tomography [17]. Circulating monocytes from patients with severe coronary atherosclerosis elicited a stronger ex vivo pro-inflammatory cytokine and chemokine response towards stimulation with LPS compared to healthy subjects without atherosclerosis [20]. Curiously, the activating H3K4me3 marks were reduced at the promoters of *TNF*, *IL*-*6*, and *IL*-*1β* in coronary symptomatic patients compared to controls. However, the repressive histone modifications H3K9me3 and H3K27me3 were depleted from promoters of genes encoding *TNF* and *IL*-*6* of monocytes from symptomatic patients. Although this epigenetic profile differs from in vitro β-glucan-trained immunity, it does show that patients with active atherosclerosis are epigenetically distinguishable from healthy controls.

## Metabolism of innate immune cells in atherosclerosis

Cellular metabolism of innate immune cells has recently emerged as an important determinant of immunological responses. These studies have given rise to the field of immunometabolism, which has broad implications for human disease, and is the subject of various recent excellent reviews [7, 8, 21]. This review will focus primarily on the immunometabolism of monocytes and macrophages and how the inner energy processing of these cells contributes to the entropy of atherosclerosis.

It is currently appreciated that cellular metabolism is not merely a source of energy for the cell. Through the breakdown of nutrients, ATP is produced which is used for the various energetically demanding processes within the cell, but the intermediate metabolites of the various intracellular metabolic pathways also serve many important biological roles in their own rights. A classic example is the divergent use of arginine by so-called classically (LPS/IFNγ) activated macrophages (formally known as M1 macrophages), and alternatively (IL-4/IL-13) activated macrophages (M2 macrophages) [22]. The pro-inflammatory M1 macrophages synthesize nitric oxide (NO) from L-arginine via inducible-nitric oxide synthase (iNOS). M1 macrophages produce NO to signal important cues including vasodilation, insulin secretion, and angiogenesis, as well as being an important microbicidal agent. The immune regulatory M2 macrophages on the other hand catabolize arginine via arginase (Arg1), producing L-ornithine in the process. L-ornithine can then be further broken down into polyamines and L-proline which can be used to support cell growth and division, as well as serving as an essential building block for collagen production contributing to wound healing and tissue repair.

Another stark difference between the metabolic activities of macrophages is the difference in their glucose usage. LPS/IFNγ-activated macrophages metabolize glucose primarily via glycolysis, while IL-4/IL-13-induced macrophages metabolize glucose via oxidative phosphorylation (OXPHOS). Glycolysis and OXPHOS diverge after pyruvate production, where in glycolysis, pyruvate is converted into lactate via lactate dehydrogenase (LDH), in total producing two molecules of ATP for each molecule of glucose, while in OXPHOS, pyruvate is shuttled into the mitochondria and enters the tri-carboxylic acid (TCA) cycle netting 32 molecules of ATP for every molecule of glucose. The reliance on glycolysis by energetically active cells seems counterintuitive at first; however, this metabolic effect is well known for its importance in driving the growth of cancer cells, termed the *Warburg* metabolic shift. Although less efficient, glycolysis can result in a rapid increase in ATP production, and, moreover, produces various important metabolites that fuel the pentose phosphate pathway (PPP) as well as fatty acid synthesis (FAS) for the production of amino acids and fatty acids vital for carrying out various cellular activities, as well as supporting cell growth and division. Cells with an archetypal Warburg effect also maintain a TCA cycle, but have two blockades after citrate and succinate resulting in the accumulation of these metabolites [23, 24]. Citrate is vital for phospholipid and cholesterol synthesis, both crucial for formation of new membranes, a central process during cell activation. In turn, succinate activates HIF-1α and induces IL-1β production [25].

**Fig. 2:**
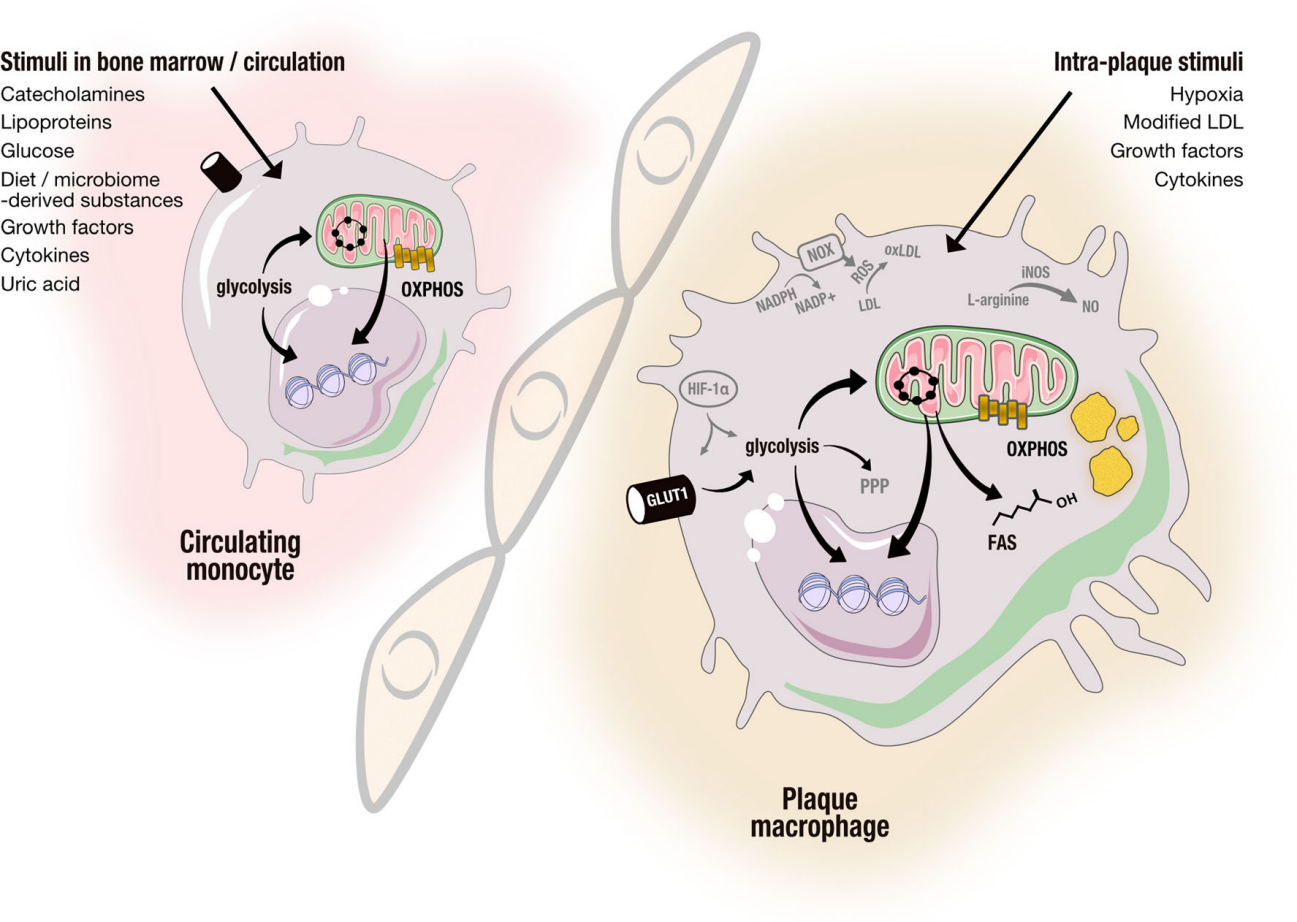
Divergent metabolic pathways in monocytes and plaque resident macrophages. Monocytes and their bone marrow progenitors are exposed to various stimuli, including lipoproteins, glucose, and diet/microbiota-derived substances that can modify the monocyte phenotype by changing the intracellular metabolism, as mentioned in the text. Once within atherosclerotic plaques, macrophage metabolism can be modified further by stimuli that are present in the atherosclerotic plaque micro-environment, including hypoxia, modified lipoproteins, and cytokines

In this review, we discuss in detail the potential role of immunometabolism in the pathophysiology of atherosclerosis. We propose changes in intracellular metabolism in circulating monocytes and bone marrow progenitors that are driven by exposure to systemic pro-atherogenic stimuli, such as lipoproteins, glucose, catecholamines, and products that are derived from the diet and gut microbiome. Once differentiated into plaque macrophages, the functional state can be further influenced by stimuli in the micro-environment of the atherosclerotic plaque, such as modified lipoproteins, and hypoxia [26]. Finally, we propose that the susceptibility for these triggers to modulate intracellular metabolism and functional state is influenced by the genetic background of these cells (Fig. 2).

## Glycolysis as a determinant of inflammation in atherosclerosis

Monocytes stimulated with inflammation-inducing stimuli, such as LPS or oxLDL, switch towards preferential glycolytic metabolism. The pro-inflammatory NF-κB regulates the expression of HIF-1α, leading to the increased expression of the GLUT-1 glucose transporter, which consequently enhances the uptake of glucose to meet increasing demand. Another important activator of the HIF-1α transcription factor is hypoxia. Atherosclerotic plaques are rich with regions of hypoxia where oxygen availability is limited. When a cell is exposed to low oxygen levels, the HIF-1α transcription factor is stabilized and initiates glycolytic metabolism, reducing the cell reliance on OXPHOS, as well as increasing the expression of the key glycolysis proteins GLUT1, hexokinase II (HK-II), and 6-phosphofructo-2-kinase/fructose-2, 6-bisphosphatase (PFKFB3), resulting in increased glycolytic flux [27]. These activated macrophages take up large quantities of glucose and begin to secrete high levels of cytokines. Indeed, mouse models of atherosclerosis have demonstrated the co-localization of hypoxia, HIF-1α expression, and FDG uptake in macrophages within the atherosclerotic plaque [27–29]. With regard to using FDG uptake as measure for glycolysis, a recent study concluded that this uptake might not reveal all differences in glycolytic rate; it appeared that stimulation of peritoneal macrophages with M-CSF or GM-CSF resulted in a 4.7- and 2.8-fold increase in glycolytic activity, whereas FDG uptake was increased to a similar extent in both situations [30].

An elegant proof-of-principle experiment showed that *Ldlr* ^−/−^ mice were less susceptible to atherosclerotic plaque formation when *HIF*-*1α* was knocked out in myeloid cells [28]. *GLUT*-*1* expression was negatively affected in these HIF-1α-deficient cells, suggesting a role for glucose metabolism in this protective knockout.

HIF-1α is also important for initiating macrophage infiltration into tissue. Underlying this increased ability to migrate was the induction of pyruvate dehydrogenase kinase isozyme 1 (PDK1) by HIF-1α [31]. PDK-1 catalyzes the conversion of pyruvate into lactate. Inhibition of glycolysis using 2-deoxy-D-glucose suppressed migration, suggesting a non-dispensable role for glucose metabolism in initiating tissue migration by macrophages in response to hypoxia.

It was recently shown that monocytes from patients with symptomatic atherosclerosis expressed higher levels of glycolysis-related genes, including *HK2*, *PFKFB3*, and *PKM1* [20]. Shirai and colleagues showed that circulating monocytes from patients with coronary artery disease had an increased appetite for glucose, and that upon ex vivo differentiation into macrophages, maintained their appetites. Pyruvate kinase M2 (PKM2) was highly upregulated in these macrophages, which phosphorylates the transcription factor STAT3 resulting in higher levels of IL-6 and IL-1β production, contributing to the inflammatory state of these cells [24]. PKM2 was also shown to stabilize the production of ROS by the mitochondria contributing the oxidative potential of these macrophages.

The origin of these circulating cells with increased glycolysis in patients with atherosclerosis remains unclear; however, there is evidence that metabolic changes with increased glycolysis are already present in hematopoietic stem and progenitor cells (HSPCs). Glucose uptake was increased in atheromatous plaques, spleens, and the bone marrow of the *ApoE* ^−/−^ mouse model of atherosclerosis [32]. The oxygen consumption and proliferation were increased in HSPCs. In addition, mitochondrial oxygen consumption rate was also higher in these cells and circulating leukocytes, distinguishing this metabolic state from the classical Warburg metabolism [32]. There were parallel increases in citrate, fumarate, and malate concentrations in circulating leukocytes. However, succinate was not increased, likely due to an increased succinate dehydrogenase expression. Studies have shown that conversion of succinate and pyruvate into the TCA cycle is essential for HPSC expansion. Mice with defective cholesterol efflux transporters ABCA1 and ABCG1 in the myeloid lineage are characterized by a massive expansion and proliferation of HSPCs [33]. Further studies revealed that intracellular cholesterol loading activates GLUT1 expression, glycolysis, and oxidative phosphorylation via IL-3Rβ/GM-CSF signaling [34].

Although glycolysis is clearly important for fueling the inflammation that drives the progression of atherosclerosis, it appears that an increased glycolytic rate in macrophages alone is not enough to induce atherosclerosis. Induced GLUT1 overexpression in myeloid cells could not induce atherosclerotic lesions in *Ldlr* ^−/−^ mice, despite having increased glycolytic flux, activated PPP, and a compensatory reduction in fatty acid β-oxidation. Importantly, overall oxygen consumption was not increased in these cells [35]. Similarly, overexpression of GLUT1 in the mouse J774 macrophage cell line showed increases in glycolysis and flux through the PPP; however, no significant increase was seen in inflammatory gene expression.

Recently, it was demonstrated that a large portion of macrophages inside the atherosclerotic plaque originate from macrophage proliferation within the plaque rather than via monocyte recruitment into the plaque and macrophage differentiation [3]. Importantly, glycolysis fuels the PPP to synthesize amino acids needed for the increased protein, RNA, and DNA synthesis burden of inflamed macrophages. Increased amino acid production by the PPP is also important for the production of nucleotides used for the escalated transcription in activated macrophages. This boost in nucleotide synthesis may be important for accommodating DNA replication needed for this enhanced cellular proliferation of activated macrophages.

Furthermore, recent evidence shows the importance of non-coding RNAs in potentiating atherosclerosis through metabolic reprogramming. Mir-33, which is triggered by a Western diet, specifically has been shown to be important in regulating a pro-inflammatory phenotype by inhibiting AMKP, which suppresses fatty acid oxidation (FAO) and enhances glycolysis [36, 37].

Another mechanism that potentially links activation of glycolysis and PPP with atherogenesis is the production of NADPH, owing to activation of the PPP. NADPH oxidases (NOX), of the mitochondria and phagosomes, remove electrons from NADPH and transfer them to oxygen molecules generating oxygen radicals that play an important role in the phagocytic degradation of invading pathogens. Oxygen radicals are also important for contributing to the oxidative stress within the atherosclerotic plaque by oxidizing proteins and fatty acids, most famously LDL molecules. An interesting observation is that only pro-inflammatory macrophages like LPS/IFNγ-activated and trained macrophages have an activated PPP [18], suggesting that PPP is vital for facilitating the increased cellular dynamics of activated macrophages, as well as integrating this inflammatory process in the pathogenesis of atherosclerosis [25].

In a series of recent studies, it has been shown that trained immunity is also critically dependent on activation of the glycolytic rate. Importantly, training with both β-glucan and BCG activates glycolytic flux in monocytes, but a classical Warburg metabolic shift is observed only in β-glucan-trained cells; stimulation with BCG results in upregulation of both glycolysis and oxidative phosphorylation [38, 39]. Arts et al. demonstrated that genetic variation in key glycolytic enzymes such as *HK2* and *PFKP* determined the magnitude of BCG-induced training, illustrating the concept that genetic variation affects the susceptibility of the innate immune system for training [38]. The causal role for glycolysis in trained immunity has been validated by the observation that trained immunity is completely prevented by pharmacological blockers of glycolysis [18]. For oxLDL-induced training, the role for glycolysis remains to be determined.

## Intracellular cholesterol metabolism and atherosclerosis

The lipid bilayer that constitutes the membranes of cells and organelles is rich in cholesterols. This cholesterol is important for maintaining the structural integrity and fluidity of the membrane as well as signal transduction. As such, cholesterol plays many important roles in facilitating the formation of immunological synapses and endocytosis of foreign bodies, as well as cellular growth and proliferation among many other cellular processes. Apart from their structural importance, isoprenoid intermediates of the cholesterol synthesis pathway are used for the prenylation of signaling molecules, which accommodate their integration into the cell membrane, allowing for the transduction of important immunological processes.

It is well known that a major risk factor for the development of atherosclerosis is the presence of high levels of modified cholesterol, most notably oxLDL. Within atherosclerotic plaques, oxLDL is taken up by macrophages resulting in cholesterol-laden macrophages often dubbed foam cells. Foam cells contribute to the pathogenesis of the plaque by secreting high amounts of pro-inflammatory cytokines and chemokines, as well as by the production of matrix metalloproteinases (MMP) which degrade the extracellular matrix of the plaque [40, 41].

Modified LDLs are highly pro-inflammatory, and as discussed previously, can induce a trained immune phenotype when stimulated for 24 h. Key metabolic pathways upregulated in macrophages trained with β-glucan or BCG include the cholesterol synthesis pathway, and inhibition of cholesterol synthesis with statins abrogate β-glucan-induced trained immunity both in vivo and in vitro [39]. The role of the cholesterol synthesis pathway for oxLDL-induced training remains to be established.

It is obvious that the control of cholesterol influx versus efflux is vitally important for the progression of atherosclerosis. At the base of this regulation are key transcription factors. Liver X receptor (LXR) increases the expression of cholesterol efflux transporters, controlling the amount of cholesterol removed from the cell. LXR activation has potent anti-inflammatory effects, at least partly due to the inhibition of TLR-2, TLR-4, and TLR-9 signaling to their downstream NF-κB and MAPK effectors via changes in membrane lipid composition through ABCA1, which disrupts the recruitment of MyD88 and TRAF6 [42]. In contrast, a previous study reported that LXR activators ameliorate atherosclerosis in *Ldlr* ^−/−^ mice independent from ABCA1 and ABCG1 in myeloid cells [43].

SREBP1c, a member of the protein SREBP family, turns on the fatty acid synthesis pathway, a pathway upregulated in pro-inflammatory LPS/INFγ-activated macrophages [44]. SREBPs are located within the ER, where they are retained by cholesterols, desmosterol, and oxysterols. SREBPs are released when intracellular concentrations of these metabolites drop critically. The released SREBPs migrate into the nucleus and drive the expression of LDL receptors as well as genes involved in the cholesterol biosynthesis pathway and the fatty acid synthesis pathway. In addition, SREBP1a is a target gene of NF-κB and are therefore induced by inflammation [45].

Rather surprisingly, it was recently reported that foam cells derived from murine peritoneal macrophages have an anti-inflammatory phenotype, due to accumulation of desmosterol [46], which contrasts with the finding that in an atherosclerotic plaque environment, foam cells have an increased inflammatory gene expression [47]. Due to a suppressed DHCR24, desmosterol is no longer converted into cholesterol, and accumulates within foam cells. Desmosterol is an important activator of LXR, resulting in the suppression of SREBP1 and 2 processing, as well as the inhibition of inflammatory gene expression. However, foam cells are also characterized by enhanced expression of pro-inflammatory cytokines. Therefore, it is likely that cues from the atherosclerotic environment, which were not present in this experimental set up, drive the pro-inflammatory phenotype of foam cells.

The importance of cholesterol homeostasis for immune function in the context of atherosclerosis is illustrated by various observations in murine models of atherosclerosis: several animal studies have reported that LXR agonists can reduce atherosclerotic plaque formation [40]. Moreover, hypercholesterolemia and impaired cholesterol efflux in myeloid cells promote atherosclerotic lesion formation by increased proliferation of HSPCs [40].

## Fatty acid oxidation and fatty acid synthesis

In a broader sense, intracellular fatty acid synthesis and oxidation play an important role in regulating the inflammatory output of the macrophage. Namely FAO is the primary source of energy production used by anti-inflammatory IL-4/IL-13-activated macrophages [21, 48]. LPS/IFNγ-activated macrophages on the other hand downregulate FAO, favoring glycolytic metabolism for their energy demands. Transport of long-chain fatty acids into the mitochondria via CPT1 induces fatty acid oxidation in macrophages. Fatty acid oxidation is supported by the gene regulatory effects of STAT6 and PPAR-γ-co-activator 1β (PGC1β), in response to IL-4, which work together to suppress inflammatory signaling [49, 50]. FAO metabolism in macrophages has been tied to these cells’ anti-inflammatory responsiveness. Induced overexpression of CPT1, predictably resulted in increased rates of FAO, and coincided with decreased production of inflammatory cytokines [51].

By contrast, fatty acid synthesis is generally associated with a pro-inflammatory macrophage phenotype [52, 53]. As discussed earlier, SRBEP1c expression lays at the base of this enhanced fatty acid synthesis pathway in LPS/IFNγ-activated macrophages; a key gene upregulated in this pathway is the multi-complex enzyme FASN. The increased fatty acid synthesis resulting from FASN expression plays an important role in the generation of pro-inflammatory LPS/IFNγ-activated macrophages [44]. The relevance of this pathway for atherosclerosis development is highlighted by the observation that macrophage-targeted deletion of *Fasn* reduces atherosclerotic plaque formation and foam cell formation in *ApoE* ^−/−^ mice, probably through inactivation of LXRα [54].

Within the atherosclerotic plaque lay many regions of hypoxia. Hypoxia, as well as NF-κB, are important activators of HIF-1α, which stimulates stearoyl-coenzyme A desaturase, an important enzyme in FAS. It has been shown that hypoxia enhances FAS while suppressing FAO, thereby promoting triglyceride-laden macrophages [55]. Specifically, stearoyl-coenzyme A desaturase (SCD) is activated under hypoxic conditions driving the synthesis of monounsaturated fatty acids from palmitic acid. Increased intracellular levels of unsaturated fatty acids (oleic acid, linoleic acid, and arachidonic acid), but not saturated fatty acids, stimulate a pro-inflammatory phenotype by upregulating IL-1α production in foam cells [56]. However, SCD deficiency in the bone marrow of *Ldlr* ^−/−^ mice sees no changes in macrophage inflammatory function or lesion size, despite having defective cholesterol efflux [57].

Under certain conditions, machinery from FAO may participate in inflammatory processes, thereby repurposing the mitochondria away from solely producing ATP. A few studies have shown that oxidized palmitate generated by CPT1A fuels mitochondrial respiration resulting in ROS production, which subsequently activates the NLRP3 inflammasome [58–60]. It was recently shown that CPT1A expression was elevated in early atherosclerotic lesions in mice [61], potentially placing this novel approach to metabolic repurposing in the development of atherosclerosis. Although this remains hypothetical, studies like these highlight the interconnectedness of various metabolic pathways in inflammatory signaling.

## Amino acid metabolism and atherosclerosis

Although often left in the shadows of glycolysis and fatty acid metabolism, the metabolism of amino acids can have some profound and important roles in inflammation contributing to vascular pathology. In the context of atherosclerosis, the metabolism of arginine, and its byproduct NO, is vitally important for the early stages of the disease [62]. Risk factors for coronary heart disease are associated with decreased bioavailability of NO and endothelial dysfunction [63]. NOS metabolism of arginine, through an oxidation of the guanidine-nitrogen terminal of L-arginine, produces NO and citrulline as a result. NO plays an important role in maintaining the homeostasis of vascular tissue by preventing the abnormal proliferation of vascular smooth muscle cells, maintaining leukocyte interaction with the vascular wall, and regulating the presentation of antigens, as well as in maintaining vascular tone and growth [64].

Inflammation within the atherosclerotic tissues gives rise to many ROS species. NO interacts with ROS, giving rise to the even more reactive nitrogen species (RNS) such as peroxynitrite [65, 66]. Apart from lowering the bioavailability of NO, conversion into RNS have other unfavorable effects on proteins in the extracellular milieu by causing protein nitration, an important sign of tissue damage. Importantly, peroxynitrite also oxidizes lipoproteins within the intima, further generating the pro-atherosclerotic oxLDL. Classically activated macrophages are well characterized as elevating their expression of the (inducible) iNOS; elegant in situ hybridization experiments have demonstrated elevated iNOS expression by macrophages within atherosclerotic lesions when compared to healthy arterial tissue [67, 68]. Foam cells within atherosclerotic tissue were shown to have elevated levels of both COX-2 and iNOS. It is likely that cross-talk between these two metabolic pathways produce high levels of peroxynitrite furthering inflammation within the atherosclerotic plaque [69].

NADPH oxidases are an important source for ROS production. Nox1 affects atherosclerosis formation, as *Nox1* genetic deletion in *ApoE* ^−/−^ mice reduces atherosclerosis [70]. Bone marrow transplantation experiments revealed that NADPH oxidase activity in myeloid cells is indeed essential for LDL oxidation in the vascular wall [71]. As previously mentioned, trained macrophages are characterized by activation of the PPP, which is important for NADPH generation. Interestingly, ROS production was upregulated in monocytes trained with BCG and oxLDL, whereas β-glucan-induced training did not induce this effect [72].

One of the more well-studied amino acids for its role in stabilizing inflammation is glutamine. Glutamine is important for the induction of IL-1 by macrophages in response to LPS stimulation [73]. Glutamine also has potential roles in macrophages microbicidal capacity by aiding in the generation of NO by feeding into the arginine synthesis pathway. Due to these potentially important roles in the immune functioning of macrophages, glutamine is hypothesized to play an important role in sepsis and burns.

Rom et al. conducted experiments to determine the atherogenicity of various amino acids in a murine macrophage-like cell line [74]. They found that glutamine had pro-atherosclerotic effects on these macrophages: macrophages supplemented with glutamine had an increased triglyceride mass as a result of enhanced triglyceride biosynthesis by increased SREBP1 and DGAT1 expression. In addition, glutamine supplementation to *ApoE* ^−/−^ mice was associated with an enhanced ROS generation in peritoneal macrophages. [74].

Glutamine feeds into the TCA cycle by direct conversion into glutamate, α-ketogluterate, and succinate semialdehyde, serving as an important determinant of IL-4 macrophage polarization [23]. This provides the substrates fumarate and succinate which replenish the broken TCA cycle. It was recently shown that macrophages trained with β-glucan have an enhanced glutaminolysis, and that this process is vital for the induction of a trained macrophage phenotype in response to β-glucan [39]. In trained macrophages, a marked increase in fumarate and succinate accumulation was noted. Intriguingly, stimulation of monocytes with an excess of fumarate for the first 24 h of the differentiation protocol also resulted in enhanced TNF and IL-6 production, and correlated with H3K4me3 epigenetic marks at the promoters of their respective genes. It was demonstrated that fumarate directly inhibits the histone demethylase KDM5 which correlated with increased training. Providing the macrophages with α-ketogluterate, the substrate for KDM5 activity, increase KDM5 availability and suppressed the fumarate trained phenotype. These experiments demonstrate the complex interplay between epigenetic, metabolic, and inflammatory pathways leading to innate immune memory. The role of glutaminolysis for oxLDL-induced training remains to be determined.

## Epigenetic memory and metabolic memory are interconnected

A crucial question is how the metabolic changes described in this review ultimately connect with gene expression and inflammatory phenotype of the cells. There is accumulating evidence that epigenetic reprogramming is at the center of this mechanism.

The Diabetes Control and Complications Trial (DCCT) and the follow-up Epidemiology of Diabetes Interventions and Complications (EDIC) study compared intensive glucose control to conventional glucose control in patients with type I diabetes mellitus [75, 76]. The goal of the DCCT trial was to determine the effect of strict glucose lowering on the development and progression of vascular complications of type I diabetes. The EDIC trial then subjected all patients to the intensive treatment plan and monitored their progression. Most striking was the observation that patients subjected to conventional treatment during DCCT developed more severe complication during the EDIC phase, when compared to patients that received intensive treatment during DCCT, despite identical treatment regimens in the follow-up period. This suggests long-term memory from the period when patients had elevated glucose levels, which has been termed *hyperglycemic memory* [77] or *legacy effect* [78]. A follow-up was conducted on a few volunteers of the EDIC trial where epigenome-wide analysis of H3 acetylation showed an increase of this mark in monocytes of patients that were treated with the convention treatment [79]. These data strongly suggest that long-term epigenetic memory may be influenced by glucose metabolism. In addition, various intermediate metabolites function as substrates or cofactors of epigenetic enzymes, which has been the subject of excellent previous reviews [77, 80].

Various metabolites serve as important substrates or cofactors for epigenetic enzymes. Histone acetyltransferase uses acetyl-CoA as the essential acetyl donor during the acetylation of histone lysine residues [81]. Acetylated histone marks, such as H3K9ac and H3K27ac, are associated with active promoters and enhancers [82, 83]. Histone deacetylases (HDACs) on the other hand decrease chromatin accessibility and inhibit gene transcription by removing acetyl groups from lysines. In this process, the metabolic cofactor NAD+ is essential for deacetylation of histones by the sirtuin family of HDACs [84]. Histone methylation is written by histone methyltransferases, which rely on S-adenosyl methionine [85]. Methylated histones serve many diverse and counter-regulatory functions within the cell. Most notably, H3K4me1, H3K4me3, and H3K36me3 are generally enriched at active promoters and enhancers [86]. By contrast, H3K9me3 and H3K27me3 are known as repressive marks due to their enrichment on silent genes. Lysine-specific histone demethylase 1A (KDM1A) erases histone methylation by relying on FAD oxidative potential [87, 88]. The histone demethylase Jumonji is dependent on the TCA intermediate α-ketoglutarate [89], adding to the pool of necessary cofactors that drive histone modification flux within the nucleus.

In the context of trained immunity, genome-wide reprogramming of H3K4me1, H3K4me3, and H3K27Ac in β-glucan-trained cells has been described [19]. Moreover, training with BCG and β-glucan as well as with oxLDL was completely prevented by co-treatment with the nonspecific histone methyltransferase inhibitor methylthioadenosine [12, 16, 18]. As mentioned before, for β-glucan-induced training, it was elucidated that fumarate accumulates from glutamine replenishment of the Krebs cycle, and in turn inhibits the KDM5 family of H3K4 demethylases, which subsequently leads to maintenance of this important epigenetic mark of open chromatin in trained monocytes [39].

## Clinical relevance and future directions

Despite optimal treatment of traditional cardiovascular risk factors with cholesterol-lowering agents and antihypertensives, a significant residual cardiovascular risk remains. Therefore, the inflammatory component of atherogenesis has recently gained interest as potential treatment target. Currently, several large clinical trials are exploring whether treatment with anti-inflammatory agents such as methotrexate and the anti-IL-1β-antibody canakinumab is able to prevent cardiovascular events in high-risk populations [90]. The further elucidation of the metabolic reprogramming in the development of atherosclerosis might aid in the development of more targeted treatment strategies to prevent or treat atherosclerosis.

Interestingly, some drugs that are already in use in clinical practice for decades, such as metformin and statins, are now described to interfere with key metabolic pathways that drive innate immune activation in the context of atherosclerosis. More specific compounds that inhibit the glycolytic rate might have the potential to limit atherosclerosis. Interestingly, partial and transient inhibition of glycolysis, even in mice in vivo, has proven to be able to limit pathological angiogenesis, a process in which activation of endothelial cell glycolysis is a major driving force [91]. Tumor endothelial cells are also characterized by a hyper-glycolytic metabolism, and blockade of the glycolytic enzyme PFKFB3 was able to reduce cancer cell invasion and metastasis [92]. These examples suggest that similar strategies might prove to be beneficial in the context of atherosclerosis.

As a separate layer of metabolic regulation, non-coding RNAs offer an exciting possible pharmacological target in the treatment of atherosclerosis. In *Ldlr* ^−/−^ mice on a Western diet, miR-33 promotes a pro-inflammatory macrophage phenotype by fueling glycolysis and repressing FAO via inhibition of AMKP [37]. This in turn represses the expression of ALDH type 2 which regulates retinoid acid synthesis, which can promote the development of anti-atherosclerotic FOXP3^+^ Tregs. Indeed, systemic treatment with miR-33 inhibitors for 8 weeks prevented this metabolic switch and profoundly reduced atherosclerosis progression.

In summary, evidence is accumulating that innate immune cell metabolism is affected by systemic pro-atherogenic factors in the circulation and bone marrow niche, and by the local atherosclerotic plaque environment, and that this shifts these cells into a pro-atherogenic phenotype that contributes to progression of atherosclerotic lesions. Further elucidation of the underlying mechanisms might provide novel markers of cardiovascular risk and novel pharmacological targets in the battle against cardiovascular diseases. This maladaptive immune function contrasts the beneficial effects of long-term activation of the innate immune system in the context of vaccination or infectious diseases. Understanding this distinction as well as the immunometabolic differences between acute inflammation and chronic metabolic disease are important avenues of investigation moving forward.
